# The endo-lysosomal–lipid axis: bidirectional interactions between membrane trafficking dysfunction and lipid metabolic disorders

**DOI:** 10.3389/fcell.2026.1860821

**Published:** 2026-07-15

**Authors:** Yao Du, Li Li, Mingtao Du, Zhi Xu, Xiaorong Zhang, Wa Cao

**Affiliations:** 1 Department of Respiratory Medicine, Affiliated Hospital of JiuJiang University, Jiujiang, Jiangxi, China; 2 Department of Intensive Care Unit, The International Peace Maternity and Child Health Hospital of China Welfare Institute, Shanghai, China; 3 Department of Pathology, Affiliated Hospital of Jiujiang University, Jiujiang, Jiangxi, China

**Keywords:** autophagy, cholesterol trafficking, endo-lysosomal system, endo-lysosomal–lipid axis, lipid metabolism, lysosomal dysfunction, metabolic disease

## Abstract

The endo-lysosomal system is a central regulator of intracellular trafficking, cargo degradation, and metabolic homeostasis. Its dynamic function is closely intertwined with lipid metabolism, forming an integrated regulatory network termed the endo-lysosomal–lipid axis. Disruption of this axis can impair endosomal maturation, lysosomal acidification, autophagic degradation, and lysosome-centered signaling pathways, resulting in defective cellular clearance and chronic inflammatory responses. Conversely, dysfunction of the endo-lysosomal system disrupts cholesterol trafficking, lipid redistribution, and macromolecular degradation, ultimately promoting secondary lipid accumulation and metabolic imbalance. In this review, we summarize the reciprocal interactions between lipid metabolism and endo-lysosomal function, with particular emphasis on membrane trafficking, lysosomal homeostasis, autophagy, membrane contact sites, and multicellular lipid clearance networks. We further discuss how these interconnected processes contribute to disease progression and highlight emerging therapeutic strategies aimed at restoring lysosomal function and lipid homeostasis. Understanding the dynamic regulation of the endo-lysosomal–lipid axis may provide new mechanistic insights into metabolic and neurodegenerative disorders and identify novel therapeutic opportunities.

## 1 Introduction

**FIGURE 1:**
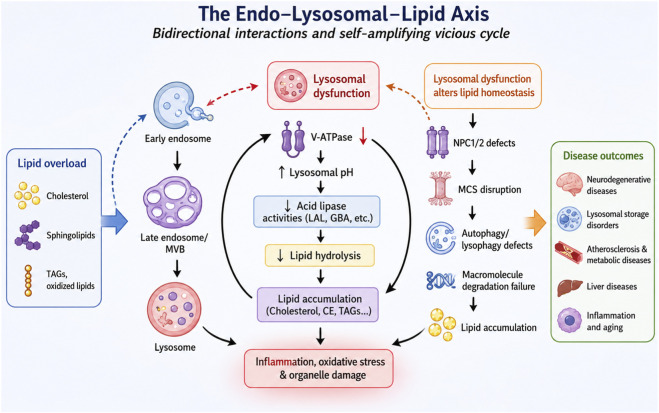
Schematic overview of the endo-lysosomal–lipid axis. Lipid overload disrupts endosomal maturation, lysosomal acidification, autophagic degradation, and inflammatory signaling. Conversely, lysosomal dysfunction impairs cholesterol trafficking, lipid clearance, and metabolic homeostasis, resulting in secondary lipid accumulation. These reciprocal interactions establish a self-reinforcing pathological cycle that contributes to metabolic and neurodegenerative diseases.

The endo-lysosomal system is a highly dynamic intracellular network responsible for membrane trafficking, cargo sorting, degradation, and recycling. Through the coordinated actions of endosomes, lysosomes, and associated trafficking machinery, this system regulates the turnover of proteins, lipids, nucleic acids, and damaged organelles, thereby maintaining cellular homeostasis. Beyond its classical degradative role, the endo-lysosomal system is now recognized as an important metabolic platform that integrates nutrient sensing, intracellular signaling, organelle communication, and stress adaptation (Yang and Wang, 2021). Lipid metabolism is another fundamental process required for cellular function. Lipids not only serve as energy reservoirs but also constitute essential structural components of biological membranes and participate in diverse signaling pathways. To maintain lipid homeostasis, cells must tightly coordinate lipid uptake, intracellular trafficking, storage, utilization, and degradation (Li et al., 2026). Among the organelles involved in these processes, endosomes and lysosomes occupy a central position. Endocytic pathways mediate the uptake and redistribution of extracellular lipids, while lysosomes participate in lipid hydrolysis, cholesterol mobilization, and autophagic degradation of lipid droplets (LDs). In parallel, membrane contact sites (MCSs) between lysosomes and other organelles facilitate non-vesicular lipid transport and metabolic communication (Thakur and O’Connor-Giles, 2025; Meyer and Kravic, 2024). Dysregulation of either lipid metabolism or endo-lysosomal function has been implicated in a broad spectrum of diseases, including neurodegenerative diseases. Notably, these pathological conditions often exhibit both lipid accumulation and endo-lysosomal abnormalities. For example, excessive cholesterol and sphingolipid accumulation can impair endosomal maturation, lysosomal acidification, and autophagic flux, whereas defects in lysosomal degradation and intracellular trafficking can disrupt cholesterol export, lipid recycling, and metabolic homeostasis. These observations suggest that lipid metabolism and endo-lysosomal biology are linked through reciprocal and self-reinforcing mechanisms rather than simple linear relationships (Radulovic et al., 2022; Roney et al., 2022). Despite increasing interest in this field, current studies are often divided between investigations of lipid metabolic pathways and those focusing on endosomal or lysosomal dysfunction. As a result, the mechanistic connections between these processes remain fragmented, and many disease-associated phenotypes cannot be fully explained by alterations in a single pathway. Moreover, emerging evidence has indicated that these interactions extend beyond individual organelles. Neuronal compartmentalization imposes unique demands on endo-lysosomal trafficking, while coordinated lipid clearance by microglia, astrocytes, macrophages, and other specialized cell types further shapes tissue homeostasis and disease susceptibility (Bhupana et al., 2025). Therefore, we propose the concept of the endo-lysosomal–lipid axis, a unified framework describing the bidirectional interactions among membrane trafficking, lysosomal homeostasis, lipid metabolism, and inflammatory signaling. Using this framework, we summarize the structural organization and trafficking dynamics of the endo-lysosomal system, discuss how lipid overload drives endo-lysosomal dysfunction and how lysosomal impairment reshapes lipid homeostasis, and highlight the importance of multicellular lipid clearance networks in disease progression. By integrating these mechanisms, this review aims to provide a comprehensive perspective on the role of the endo-lysosomal–lipid axis in metabolic and neurodegenerative diseases and identify potential avenues for therapeutic intervention (Figure 1).

## 2 Structural organization and trafficking dynamics of the endo-lysosomal system

### 2.1 Endosomal maturation and lysosomal functional organization

Endosomes are membrane-bound vesicular compartments. Based on their maturation status, they are generally classified into early endosomes (EEs), recycling endosomes (REs), or late endosomes (LEs). EEs serve as the primary sorting station of the endocytic pathway and are characterized by the enrichment of Rab5 and early endosomal antigen 1 (EEA1). During maturation, EEs undergo progressive membrane remodeling and Rab5-to-Rab7 conversion, ultimately generating LEs (Ott et al., 2025). A hallmark of LE maturation is the formation of intraluminal vesicles (ILVs) through the inward budding of the limiting membrane, resulting in the generation of multivesicular bodies (MVBs), which represent a specialized morphological state of late endosomes. Upon fusion with lysosomes, cargos enclosed within ILVs are degraded and recycled, thereby contributing to cellular homeostasis. Alternatively, fusion of MVBs with the plasma membrane leads to exosome secretion and intercellular communication (Singh et al., 2025; Zanellati and Cohen, 2022). Beyond its canonical role in cargo trafficking, endosomal maturation has emerged as a critical determinant of intracellular lipid distribution and metabolic regulation. Lysosomes represent the terminal degradative compartments of the endo-lysosomal pathway. Through fusion with LEs, autophagosomes, and phagosomes, lysosomes receive a wide variety of substrates, which are subsequently degraded and recycled for cellular reuse. Structurally, lysosomes are membrane-bound organelles enriched with a broad repertoire of hydrolytic enzymes capable of degrading diverse macromolecules. The coordinated activity of these enzymes enables the efficient turnover of intracellular components and prevents the accumulation of potentially toxic substrates (Yang and Wang, 2021; Saftig and Klumperman, 2009). In addition to their degradative role, lysosomes participate in membrane recycling and organelle quality control through their close functional association with endosomes and autophagic compartments. Recent studies have expanded the traditional view of lysosomes as simple degradative organelles. They are now recognized as metabolic hubs that integrate nutrient availability, intracellular trafficking, and cellular adaptation (Dharmasivam and Kaya, 2025). The lysosomal membrane serves as an important signaling platform where nutrient-sensing pathways coordinate anabolic and catabolic responses according to cellular metabolic status. Through these functions, lysosomes contribute not only to substrate degradation but also to the regulation of energy balance, cellular stress responses, and metabolic homeostasis. Because of their central position at the intersection of degradation, recycling, and signaling pathways, lysosomes are particularly sensitive to disturbances in intracellular metabolism. Consequently, defects in lysosomal function can influence multiple cellular processes simultaneously, linking abnormalities in membrane trafficking, autophagy, and lipid metabolism (Nixon and Rubinsztein, 2024; Ballabio and Bonifacino, 2020; Chavan and Bhattacharjee, 2025). The mechanisms by which lipid overload disrupts lysosomal homeostasis and initiates pathological signaling cascades are discussed in the following section.

### 2.2 Neuronal endo-lysosomal trafficking pathways and spatial compartmentalization

The endo-lysosomal system exhibits pronounced spatial organization within cells, reflecting the dynamic coordination between cargo sorting, intracellular transport, and degradation. In most cell types, EEs are predominantly distributed in peripheral regions, where they mediate cargo internalization and initial sorting, whereas LEs and lysosomes are enriched in the perinuclear region, facilitating cargo degradation and recycling. This spatial arrangement enables efficient coupling between membrane trafficking and lysosomal degradation. The importance of spatial compartmentalization becomes particularly evident in highly polarized cells such as neurons (Liao et al., 2019; Rajgor et al., 2021). Due to their extraordinary morphology, neurons rely on long-distance transport to maintain intracellular homeostasis. Endocytic vesicles, LEs, and autophagosomes generated in distal axons must undergo retrograde transport along microtubules toward the soma, where mature degradative lysosomes are primarily concentrated. Consequently, successful cargo clearance depends not only on endosomal maturation and lysosomal function, but also on the integrity of the intracellular transport machinery (Nixon, 2024). Recent studies have further highlighted the complexity of lysosomal transport regulation in neurons. Beyond classical motor proteins, KIF1C has recently been identified as an unconventional regulator of dynein-driven lysosomal retrograde transport. Mechanistically, KIF1C interacts with the dynein adaptor Hook3, which associates with the lysosomal protein RUFY3, thereby promoting perinuclear localization of lysosomes and efficient cargo degradation in autophagic and endocytic pathways. Interestingly, this function is largely independent of KIF1C motor activity, revealing a non-canonical mechanism that coordinates lysosomal positioning and degradative capacity. These findings further emphasize that disruption of the lysosomal transport machinery may compromise neuronal homeostasis and contribute to neurodegenerative vulnerability (Saji et al., 2024). In contrast, the soma contains the highest density of mature lysosomes and serves as the principal degradative hub of the neuron. Synaptic regions further require rapid endosomal recycling to sustain neurotransmitter receptor turnover and membrane homeostasis. These compartment-specific functions render neurons particularly vulnerable to disruptions in endosomal trafficking and lysosomal transport. Importantly, impaired retrograde transport is increasingly recognized as an early pathogenic event in multiple neurodegenerative disorders (Amin and Taverna, 2025; Tammineni and Cai, 2017). Defects in Rab7-dependent maturation, motor protein function, or lysosomal positioning compromise cargo delivery to degradative compartments, leading to the accumulation of damaged proteins, dysfunctional organelles, and undegraded lipids in distal neuronal processes. Such transport defects not only impair cellular quality control but also amplify lysosomal stress and metabolic dysfunction (Zheng et al., 2025). In Alzheimer’s disease (AD) models, reduced Rab7 activity is associated with impaired retrograde transport of autophagosomes, resulting in their accumulation within axons and defective cargo clearance. Recent studies have identified adaptor protein complex 2 subunit alpha 1 (AP2A1) as an upstream regulator of Rab7 activation. AP2A1 interacts with and activates Rab7, promoting the recruitment of the dynein-associated proteins DIC1 and RILP, thereby facilitating the retrograde axonal transport of autophagosomes. Restoration of AP2A1 expression enhances autophagic flux, reduces Aβ accumulation, and improves cognitive function in APP/PS1 mice (Wang Y. et al., 2025). In Parkinson’s disease, lysosomal dysfunction promotes α-synuclein aggregation and disrupts axonal homeostasis (Morris et al., 2024). Similarly, defective endo-lysosomal trafficking has been implicated in amyotrophic lateral sclerosis and other axonopathies (Liu et al., 2025). These observations suggest that spatial compartmentalization not only determines neuronal physiology but also shapes selective vulnerability to neurodegeneration. The spatial compartmentalization of the endo-lysosomal system highlights the intimate relationship between membrane trafficking, intracellular transport, and degradative capacity. The unique dependence of neurons on long-distance transport further underscores why disturbances in endo-lysosomal dynamics frequently contribute to neurodegeneration and metabolic imbalance (Zhou L. et al., 2025).

## 3 Reciprocal regulation within the endo-lysosomal–lipid axis

### 3.1 Membrane lipid remodeling disrupts endosomal cargo sorting

The endo-lysosomal system serves as a central platform for intracellular lipid trafficking and membrane turnover. Under physiological conditions, coordinated vesicular transport mediated by Rab GTPases, SNARE proteins, and the endosomal sorting complex required for transport (ESCRT) machinery ensures efficient cargo sorting, membrane remodeling, and lipid redistribution (Ott et al., 2025; Jahn et al., 2024). Increasing evidence suggests that alterations in membrane lipid composition are not merely a consequence of endo-lysosomal dysfunction but can also actively disrupt endosomal trafficking. Among the trafficking regulators affected by lipid imbalance, the ESCRT machinery appears to be particularly vulnerable to membrane lipid remodeling. ESCRT-dependent membrane invagination and ILV formation require precise membrane curvature and lipid organization. Cholesterol and ceramide accumulation can interfere with ESCRT assembly and membrane deformation, resulting in defective cargo sequestration and impaired MVB biogenesis. Consequently, receptors, membrane proteins, and undegraded substrates accumulate within late endosomal compartments, promoting progressive trafficking failure (Wang Y. et al., 2024; Hurley et al., 2025). Furthermore, studies have indicated that lipid transfer proteins are closely integrated with the endosomal sorting machinery. Vacuolar protein sorting 13 (Vps13), a lipid transport protein localized at ER–endosome contact sites, forms hydrophobic channels that facilitate bulk lipid transfer between organelles. The loss of Vps13 disrupts endosomal membrane lipid composition and compromises ESCRT activity, highlighting the dependence of membrane trafficking on local lipid homeostasis (Suzuki et al., 2024). Similarly, the phospholipid scramblase Any1 cooperates with Vps13 to regulate phospholipid redistribution across endosomal bilayers. A deficiency of arrestin domain-containing protein 1 (Any1) reduces MVB formation and causes the accumulation of immature endosomal intermediates, further supporting the concept that coordinated lipid transfer and membrane remodeling are prerequisites for efficient endosomal maturation (Schwabl et al., 2025; Gao et al., 2025). These findings suggest that membrane lipid remodeling constitutes an upstream driver of endosomal trafficking dysfunction. Rather than being passive cargo, lipids actively shape membrane architecture and determine the efficiency of endosomal maturation and cargo sorting. Therefore, persistent lipid accumulation initiates a pathological cascade characterized by impaired ESCRT function, defective MVB biogenesis, and progressive disruption of the endo-lysosomal trafficking network, establishing the first mechanistic layer of the endo-lysosomal–lipid axis (Reinisch et al., 2025; Tang et al., 2026).

### 3.2 Lysosomal deacidification and the self-amplifying lipid storage loop

Lysosomal acidification represents a central regulatory node within the endo-lysosomal–lipid axis and provides a mechanistic bridge linking lipid overload to metabolic dysfunction. The V-ATPase maintains an acidic lysosomal lumen that supports hydrolase activity, cholesterol mobilization, membrane trafficking, and autophagic degradation (Zhou N. et al., 2025; Mindell, 2012). However, chronic lipid overload progressively disrupts this homeostatic network. The accumulation of cholesterol and cholesteryl esters within lysosomal membranes alters membrane biophysical properties and destabilizes V-ATPase assembly, resulting in lysosomal deacidification. Elevated lysosomal pH suppresses the activity of acid-dependent enzymes such as lysosomal acid lipase (LAL), thereby impairing the hydrolysis of cholesteryl esters and triglycerides. Consequently, undegraded lipids accumulate further within lysosomes, leading to additional membrane cholesterol enrichment and further destabilization of V-ATPase. This establishes a self-amplifying lipid storage loop in which lipid accumulation and lysosomal dysfunction reinforce one another. Importantly, this process extends beyond defective degradation, as persistent lysosomal stress subsequently triggers inflammatory signaling, impairs autophagy, and metabolic reprogramming, propagating dysfunction across the entire endo-lysosomal–lipid axis (Tie et al., 2026; Hao et al., 2025; Ahn et al., 2025). Emerging experimental evidence further supports a causal relationship between lysosomal dysfunction and lipid accumulation rather than a simple association. In Niemann–Pick disease, Niemann–Pick type C1 (NPC1) deficiency directly causes lysosomal cholesterol sequestration, impaired lysosomal acidification, and defective autophagic flux (Chen et al., 2026). Similarly, restoration of lysosomal acidification in experimental models of metabolic disease rescues autophagy and alleviates hepatic steatosis (Zeng et al., 2023). Moreover, pharmacological activation of AMP-activated protein kinase (AMPK) preserves lysosomal acidification and cellular homeostasis under lipotoxic conditions. A recent study has demonstrated that when a highly differentiated primary proximal tubular epithelial cell (PTEC) model was exposed to palmitic acid, lysosomal acidification was impaired, subsequently leading to accumulation of autophagic vacuoles and activation of lysosomal biosynthesis. However, when the AMPK activity decline induced by palmitic acid was blocked by an AMPK activator, the lysosomal acidification and the differentiation profile of PTECs were preserved (Pierre et al., 2025). Moreover, persistent acidification defects also compromise lysosomal membrane integrity. Cholesterol-enriched lysosomal membranes exhibit reduced fluidity and increased susceptibility to membrane permeabilization, facilitating the leakage of lysosomal contents into the cytoplasm. Release of cathepsins and other lysosomal enzymes subsequently activates inflammatory signaling pathways, particularly the NLRP3 inflammasome. Cholesterol crystals, oxidized low-density lipoproteins (LDLs), and lipid-derived danger signals further amplify this response through mitochondrial dysfunction and reactive oxygen species generation (Ebner et al., 2023; Radulovic et al., 2026; Qi Z. et al., 2024). Collectively, these findings support the concept that lysosomal dysfunction acts not only as a downstream consequence of lipid overload, but also as an active driver of progressive metabolic remodeling. Restoration of lysosomal pH homeostasis not only improves degradative capacity but may also interrupt the vicious cycle linking lipid accumulation, lysosomal dysfunction, and chronic inflammation, highlighting lysosomal acidification as a promising therapeutic target in metabolic disease (Zeng et al., 2023; Cheong et al., 2025).

### 3.3 Autophagic failure and lipophagy defects under lipid stress

Autophagy is a fundamental, lysosome-dependent degradation pathway. Among its various forms, lipophagy represents a specialized process in which LDs are selectively delivered to lysosomes for degradation, thereby coupling intracellular lipid storage to energy utilization and metabolic regulation. Efficient lipophagy requires coordinated autophagosome formation, cargo recognition, lysosomal fusion, and hydrolytic degradation (Laval and Ouimet, 2023). LDs function as dynamic lipid reservoirs composed primarily of TGs and CEs. During nutrient deprivation or metabolic stress, LDs are engulfed by autophagosomes and delivered to lysosomes, where LAL hydrolyzes stored neutral lipids into free fatty acids and cholesterol. This process not only prevents excessive lipid accumulation but also supports cellular energy homeostasis (Zhao et al., 2026). Accumulating evidence suggests that chronic lipid overload progressively impairs autophagic flux at multiple levels. Excessive intracellular accumulation of cholesterol, CEs, and LDs increases lysosomal burden and compromises autophagosome–lysosome fusion, thereby reducing degradative efficiency. As lysosomal lipid storage increases, the capacity of lysosomes to process newly delivered substrates declines, resulting in the accumulation of undegraded autophagic cargo. Consequently, defects in lipid degradation and autophagic clearance reinforce one another, establishing a self-amplifying cycle of lysosomal overload and metabolic dysfunction (Minami et al., 2023; Wang Q. et al., 2024). Recent studies have identified several molecular regulators that directly connect lysosomal lipid degradation with autophagic activity. Spartin has been proposed as a critical regulator of LAL trafficking and lysosomal localization. Loss of spartin disrupts LAL recruitment and activity, resulting in impaired hydrolysis of TGs and CEs and promoting secondary lysosomal lipid accumulation. Importantly, lipid-laden lysosomes exhibit reduced fusion capacity with autophagosomes, further suppressing autophagic flux and accelerating intracellular lipid storage. These observations suggest that defective lipolysis and impaired autophagy are mechanistically intertwined rather than independent pathological events (Wan et al., 2024). In addition to lysosomal degradation defects, excessive lipid accumulation also interferes with autophagosome biogenesis. Experimental studies have demonstrated that ATG14 is a critical regulator of lipophagy. By directly targeting LDs and facilitating their autophagic turnover, ATG14 links autophagosome biogenesis to lysosomal lipid degradation. ATG14 deficiency results in impaired lipophagy and excessive lipid accumulation, further aggravating metabolic dysfunction (Yuan et al., 2024). These findings support a model in which chronic lipid stress drives progressive autophagic failure. Impaired lipophagy promotes lipid accumulation, whereas lipid accumulation further suppresses autophagic degradation, establishing a vicious cycle that amplifies lysosomal dysfunction and metabolic imbalance. This pathological interaction represents a critical mechanistic link within the endo-lysosomal–lipid axis and contributes substantially to the development of metabolic diseases.

### 3.4 The mechanistic target of rapamycin 1–transcription factor EB signaling hub under chronic lipid stress

In response to lysosomal stress and impaired autophagy, cells activate adaptive signaling pathways to restore degradative capacity and metabolic homeostasis. Among these regulatory networks, the mechanistic target of rapamycin 1 (mTORC1)–transcription factor EB (TFEB) axis functions as a central coordinator of lysosomal biogenesis and metabolic adaptation. mTORC1 signaling promotes anabolic processes and suppresses autophagic degradation, whereas TFEB activation stimulates the expression of genes involved in lysosomal biogenesis, membrane trafficking, autophagy, and substrate turnover. Through the coordinated regulation of these pathways, the mTORC1–TFEB axis maintains cellular degradative and metabolic homeostasis (Ebner et al., 2023; Panwar et al., 2023). This finding has been confirmed using cells subjected to glucolipotoxicity, as well as in a mouse model treated with streptozotocin and fed a high-fat diet. Excessive intracellular accumulation of cholesterol and other lipotoxic species alters lysosomal nutrient sensing and contributes to persistent dysregulation of mTORC1 signaling. As a consequence, TFEB remains sequestered in the cytoplasm and fails to activate the transcriptional programs required for lysosomal renewal and autophagic adaptation (Ai et al., 2025). The pathological significance of this signaling imbalance extends beyond reduced lysosome numbers. Suppression of TFEB-dependent gene expression limits lysosomal biogenesis, decreases degradative capacity, and compromises cellular responses to metabolic stress. Consequently, lipid accumulation cannot be effectively cleared, leading to further lysosomal burden and progressive deterioration of cellular homeostasis (Wang H. et al., 2025). Importantly, dysfunction of the mTORC1–TFEB axis establishes another layer of reciprocal regulation within the endo-lysosomal–lipid axis. Lipid overload suppresses lysosomal adaptive responses through mTORC1–TFEB dysregulation, whereas impaired lysosomal renewal further aggravates lipid accumulation and metabolic stress. This signaling circuit therefore acts in concert with trafficking defects, lysosomal deacidification, and autophagic failure to drive the progression of metabolic and neurodegenerative diseases (Panwar et al., 2023; Seo et al., 2026).

## 4 Consequences of lysosomal dysfunction on lipid homeostasis

### 4.1 Lysosomal control of lipid trafficking and redistribution

Beyond their role in degradation, dysfunctional lysosomes exert a profound effect on intracellular lipid trafficking and metabolic homeostasis. Once lysosomal acidification and degradative capacity are compromised, defects in cholesterol export and lipid redistribution emerge as major downstream consequences. These alterations not only promote lysosomal lipid storage but also initiate broader metabolic adaptations that extend throughout the cell (Chen L. et al., 2024). Following LDL endocytosis, CEs are hydrolyzed by LAL, generating free cholesterol within the lysosomal lumen. This cholesterol is subsequently transferred by Niemann–Pick type C2 (NPC2) to the lysosomal membrane protein NPC1, which mediates cholesterol export to other intracellular compartments, including the endoplasmic reticulum (ER), plasma membrane, and mitochondria (Pfrieger, 2023; Appu et al., 2025). Under conditions of lipid overload, lysosomal cholesterol export becomes progressively impaired. Defects in NPC1/NPC2-mediated transport promote cholesterol sequestration within late endosomes and lysosomes, resulting in the characteristic phenotype of lysosomal lipid storage. Importantly, cholesterol retention within lysosomes not only disrupts membrane composition but also alters the intracellular distribution, thereby affecting multiple metabolic pathways (Appu et al., 2025; Ndoj et al., 2025). Recent evidence has suggested that microglial lipid dysregulation actively contributes to disease progression rather than representing a secondary response to neuronal injury. In a myeloid cell-specific NPC1-deficient mouse model, loss of NPC1 induced profound alterations in microglial lipid homeostasis, accompanied by microglial hyperactivation, neuroaxonal pathology, astrogliosis, and elevated neurofilament light chain levels. These findings indicate that impaired lipid processing in microglia can directly promote neurodegeneration and disease progression (Dinkel et al., 2024). In addition, the lysosomal calcium channel TRPML1 regulates endo-lysosomal fusion, membrane trafficking, and lipid clearance via calcium-dependent signaling pathways. Excess cholesterol accumulation suppresses TRPML1 activity despite preserved channel expression, resulting in defective lysosomal fusion and impaired cholesterol turnover (Qi J. et al., 2024; Hu et al., 2024). Disturbances in cholesterol export and lysosomal calcium signaling reinforce one another, promoting progressive lipid accumulation and metabolic imbalance. Rather than acting as a passive storage compartment, the lysosome actively governs the intracellular distribution of lipids (Jan and Jan, 2025). Importantly, defects in lysosomal cholesterol export not only result in local lipid accumulation but also alter cholesterol availability in other organelles. Because the endoplasmic reticulum serves as the primary cellular cholesterol sensor, impaired cholesterol redistribution can profoundly influence metabolic signaling pathways. This inter-organelle communication is largely coordinated through membrane contact sites, which couple lysosomal lipid handling to cellular metabolic adaptation (Gómez-Sánchez et al., 2025).

### 4.2 MCSs and non-vesicular lipid transport

Although vesicular trafficking is essential for intracellular lipid movement, increasing evidence indicates that a substantial proportion of lipid redistribution occurs through non-vesicular transport at MCSs. These specialized structures enable the close apposition of organelle membranes without membrane fusion, thereby facilitating rapid and highly regulated lipid exchange (Calì et al., 2025). Among the various MCSs identified to date, contacts between lysosomes and the ER have emerged as critical regulators of cholesterol homeostasis (Gómez-Sánchez et al., 2025). At these sites, lipid transfer proteins shuttle cholesterol and phospholipids directly between organelles, allowing cells to rapidly adjust their membrane composition and metabolic status in response to changing nutrient conditions. Oxysterol-binding protein-related protein 5 (ORP5) is a key mediator of lipid exchange at ER–lysosome contact sites. ORP5 simultaneously interacts with phosphoinositides on lysosomal membranes and phosphatidylinositol-4-phosphate on the ER membrane, forming a molecular bridge that facilitates non-vesicular cholesterol transport. Through this mechanism, cholesterol released from lysosomes is efficiently delivered to the ER, thereby preventing lysosomal cholesterol accumulation and maintaining intracellular lipid balance. Importantly, cholesterol transport through ER–lysosome contact sites is not merely a redistribution process but also serves as a critical signaling event (Tan and Finkel, 2022; Sarkar et al., 2025). The ER functions as a major cholesterol-sensing organelle, where fluctuations in cholesterol availability regulate the sterol regulatory element-binding protein (SREBP) pathway. Under cholesterol-replete conditions, the SCAP–SREBP complex is retained in the ER through interaction with Insig proteins, preventing transcriptional activation. Conversely, reduced ER cholesterol levels promote the translocation of the SCAP–SREBP complex to the Golgi apparatus, where proteolytic processing releases the active SREBP transcription factor, stimulating the expression of genes involved in cholesterol synthesis and lipid metabolism (Kober et al., 2021; Li et al., 2025; Liu et al., 2021). Disruption of ORP5-dependent cholesterol transport uncouples lysosomal cholesterol storage from ER cholesterol sensing. As a consequence, cholesterol becomes trapped within endo-lysosomal compartments, while the ER incorrectly senses intracellular cholesterol levels as insufficient, resulting in inappropriate activation of the SREBP pathway. This mismatch between cholesterol distribution and cholesterol sensing promotes excessive lipid synthesis and further aggravates metabolic dysfunction (Chen J. et al., 2024; Naito et al., 2023). Emerging evidence has further suggested that cholesterol sensing is closely integrated with fatty acid and triglyceride metabolism. Diacylglycerol acyltransferase 2 (DGAT2), a key enzyme involved in triglyceride synthesis, regulates ER phosphatidylethanolamine homeostasis and indirectly modulates SREBP-1 activation. Experimental studies have demonstrated that DGAT2 deficiency suppresses SREBP-1 processing and lipogenic gene expression, indicating that lipid synthesis pathways and cholesterol-sensing mechanisms are tightly interconnected. Together, these findings suggest that disturbances in membrane contact site-mediated lipid transport can extend beyond lysosomal cholesterol accumulation to drive broader metabolic reprogramming (Rong et al., 2024). Rather than functioning solely as structural interfaces between organelles, ER–lysosome contact sites act as regulatory hubs integrating lipid transport with metabolic signaling. Restoration of contact site-mediated cholesterol trafficking may represent a promising strategy for correcting both lysosomal lipid storage and downstream metabolic abnormalities (Tan and Finkel, 2022).

### 4.3 Convergence of macromolecular storage disorders on the endo-lysosomal-lipid axis

**FIGURE 2:**
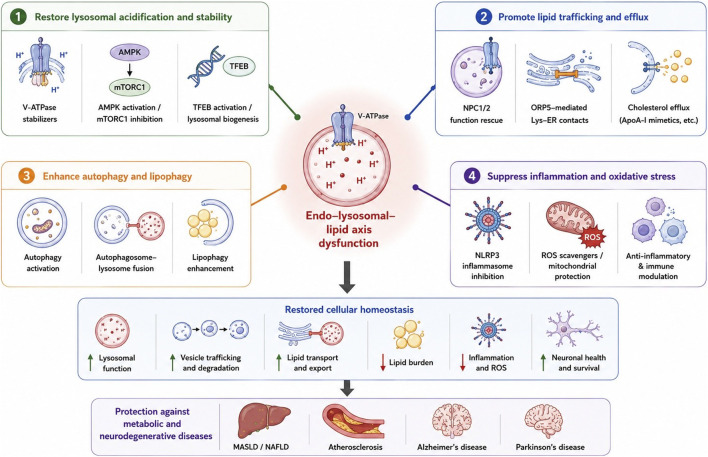
Therapeutic targeting of the endo-lysosomal–lipid axis. Therapeutic interventions targeting distinct nodes of the endo-lysosomal–lipid axis may restore cellular homeostasis. These strategies include stabilization of lysosomal acidification, enhancement of cholesterol trafficking and membrane contact site function, activation of autophagy and lipophagy, and suppression of inflammatory signaling. By simultaneously correcting defects in degradation, lipid transport, and stress responses, these interventions may interrupt the self-amplifying pathological cycle linking lysosomal dysfunction to lipid accumulation, thereby limiting the progression of metabolic and neurodegenerative diseases.

Lysosomes are responsible for the degradation of a broad spectrum of biological macromolecules, including proteins, nucleic acids, polysaccharides, and lipids. Although these substrates are often investigated independently, extensive evidence indicates that defects in the degradation of non-lipid macromolecules frequently culminate in secondary disturbances in lipid homeostasis (Settembre and Perera, 2024). Rather than representing isolated pathological events, macromolecular storage and lipid accumulation commonly coexist within dysfunctional lysosomes and mutually reinforce one another. One central mechanism underlying this interaction is the progressive occupation of lysosomal degradative capacity by undegraded substrates (Parenti et al., 2021). The accumulation of protein aggregates, nucleic acid fragments, or glycosaminoglycans (GAGs) increases the lysosomal burden and limits the availability of hydrolases, membrane transporters, and the trafficking machinery required for lipid processing. As lysosomes become overloaded, cholesterol export, lipid hydrolysis, and autophagic degradation gradually decline, creating a permissive environment for secondary lipid deposition (Calhoon et al., 2025; Xu et al., 2024). Protein degradation defects provide a representative example of this process. Impaired lysosomal proteolysis promotes the accumulation of misfolded proteins, including α-synuclein, which is closely associated with Parkinson’s disease and related neurodegenerative disorders (Morris et al., 2024). Increasing evidence suggests that cholesterol metabolites, such as 27-hydroxycholesterol, further accelerate α-synuclein aggregation, whereas protein accumulation exacerbates lysosomal dysfunction and impairs intracellular lipid handling (Dai et al., 2023). Similarly, defects in endosome-to-lysosome trafficking can delay the degradation of signaling receptors, such as the epidermal growth factor receptor (EGFR), resulting in the persistent activation of metabolic and inflammatory pathways that promote hepatic lipid accumulation and metabolic dysfunction (Song et al., 2021). Moreover, defects in nucleic acid degradation represent another important source of lysosomal stress. Failure to eliminate cytosolic DNA and RNA activates innate immune pathways, particularly the cGAS–STING signaling axis, leading to sustained type I interferon production and chronic inflammation. Prolonged activation of this pathway disrupts lipid metabolic adaptation, impairs adipose tissue function, and aggravates hepatic steatosis, thereby indirectly contributing to lipid metabolic imbalance (Hou et al., 2023; Xu et al., 2025). These observations suggest that lipid dysregulation often represents a downstream consequence of impaired macromolecular clearance rather than a primary metabolic defect. Importantly, recent therapeutic studies have further supported the mechanistic link between macromolecular degradation and lipid homeostasis. Pharmacological inhibition of EGFR signaling partially reverses lipid accumulation and inflammation associated with defective endo-lysosomal trafficking (Xu et al., 2026). Similarly, suppression of cGAS–STING signaling alleviates inflammation-driven metabolic dysfunction, while restoration of lysosomal enzyme activity through enzyme replacement therapy, gene therapy, or CRISPR-engineered cell therapies effectively reduces substrate storage and improves lysosomal function in experimental models of lysosomal storage disorders (Medoh and Abu-Remaileh, 2024; Li et al., 2020). Although these approaches target distinct molecular pathways, they converge on a common outcome—the restoration of lysosomal processing capacity and the correction of secondary lipid dysregulation. Collectively, these findings support a unifying concept in which defects in protein, nucleic acid, or polysaccharide degradation converge on a common pathological endpoint characterized by lysosomal overload, impaired intracellular trafficking, and secondary lipid deposition (Ebner et al., 2025). This convergence highlights how disturbances in diverse degradative pathways collectively destabilize the endo-lysosomal–lipid axis and identifies the restoration of lysosomal homeostasis as a promising therapeutic strategy for metabolic and neurodegenerative diseases (Figure 2).

## 5 Multicellular lipid clearance networks

### 5.1 Professional phagocytes in lipid clearance

Although intracellular lipid degradation is primarily governed by the endo-lysosomal system, maintenance of tissue lipid homeostasis requires the efficient clearance of extracellular lipid debris and damaged cellular components. Professional phagocytes, particularly macrophages in peripheral tissues and microglia in the central nervous system, play indispensable roles in this process by coupling lipid uptake to lysosomal degradation (Ebner et al., 2023). Macrophages continuously internalize oxidized lipoproteins, apoptotic bodies, and lipid-rich cellular debris through scavenger receptors such as CD36. Chronic lipid overload overwhelms lysosomal degradative capacity, resulting in Cholesteryl ester(s) accumulation and foam cell formation, a hallmark of atherosclerotic lesions (Tang et al., 2026; Li et al., 2023). These findings indicate that lysosomal processing efficiency within macrophages represents a critical determinant of tissue lipid homeostasis. Microglia perform analogous functions within the central nervous system. Beyond immune surveillance, they actively participate in myelin turnover, synaptic remodeling, and clearance of extracellular lipid aggregates (Tabor et al., 2026). Recent studies have identified triggering receptor expressed on myeloid cells 2 (TREM2) as a central regulator of microglial lipid sensing and metabolism. Activation of TREM2 promotes phagocytosis, lysosomal lipid degradation, and metabolic adaptation, whereas TREM2 deficiency results in intracellular lipid droplet accumulation, impaired lysosomal function, and chronic neuroinflammation. Lipid droplet-accumulating microglia have been consistently observed in aging and neurodegenerative disorders, highlighting the importance of microglial lipid clearance in maintaining neuronal homeostasis. Importantly, the emerging success of TREM2-targeted interventions further emphasizes the therapeutic potential of enhancing tissue-level lipid clearance. Experimental activation of TREM2 improves lipid processing, restores microglial metabolic fitness, and attenuates neuroinflammatory responses, suggesting that modulation of phagocyte-mediated lipid clearance may complement intracellular lysosome-targeted therapies (Zhong et al., 2024; Filipello et al., 2023).

### 5.2 Metabolic coupling between glial and parenchymal cells

**FIGURE 3:**
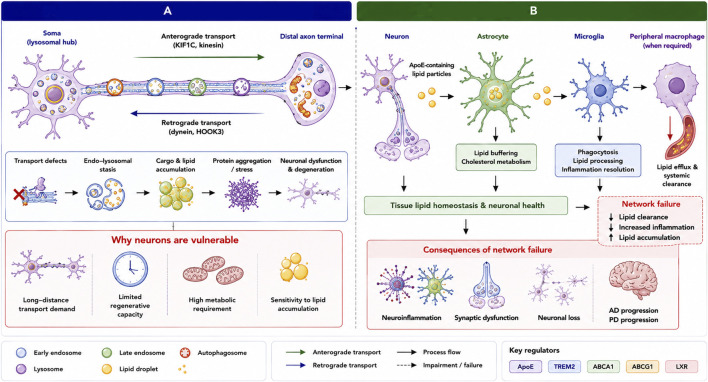
Spatial and multicellular regulation of the endo-lysosomal–lipid axis in the nervous system. Panel **(A)** illustrates neuronal compartmentalization of the endo-lysosomal system. Endosomes, autophagosomes, and lysosomes generated in distal axons undergo long-distance transport toward the soma, where mature degradative lysosomes are enriched. Impairment of axonal transport disrupts cargo clearance, leading to lipid accumulation, protein aggregation, and neuronal dysfunction. The unique dependence of neurons on long-range trafficking contributes to their selective vulnerability in neurodegenerative diseases. Panel **(B)** depicts the neuro-glial lipid clearance network, in which astrocytes buffer excess lipids released from neurons, whereas microglia and peripheral phagocytes mediate lipid processing, phagocytosis, and cholesterol efflux. Failure of this multicellular clearance system promotes chronic neuroinflammation and accelerates the progression of neurodegenerative disorders.

Increasing evidence has suggested that lipid homeostasis is not maintained by individual cells in isolation, but rather relies on extensive metabolic cooperation among neighboring cell populations. Accordingly, disturbances in lipid handling within one cell type can propagate metabolic stress throughout the tissue microenvironment (Ebner et al., 2023). Within the central nervous system, astrocytes play a pivotal role in neuronal lipid homeostasis. Because neurons possess limited capacity for long-term lipid storage, excess fatty acids and oxidized lipids generated during neuronal activity are frequently transferred to astrocytes for storage or degradation (Jung et al., 2023). Emerging evidence has indicated that APOE participates directly in astrocytic lipid droplet homeostasis. Under lipogenic conditions, APOE can localize to the surface of LDs and regulate their size and composition. APOE depletion or expression of the APOE4 variant results in enlarged LDs enriched in unsaturated TGs, accompanied by impaired lipid turnover and increased sensitivity to lipid peroxidation. These findings suggest that astrocytic lipid handling contributes to susceptibility to neurodegenerative disease through the regulation of intracellular lipid storage and oxidative stress (Windham et al., 2024). A similar cooperative relationship exists in peripheral metabolic tissues. In the liver, hepatocytes and Kupffer cells coordinate lipid metabolism and inflammatory responses through reciprocal communication. Kupffer cells remove damaged cellular components and excess lipids through lysosome-dependent pathways, whereas hepatocytes regulate systemic lipid production and utilization. Persistent metabolic stress disrupts this partnership and promotes the progression of metabolic dysfunction-associated steatotic liver disease (Jeelani et al., 2024). The recognition of lipid metabolism as a multicellular process has important therapeutic implications. Beyond targeting intracellular lipid degradation pathways, emerging strategies aim to restore intercellular lipid transport and clearance networks. Modulation of ApoE-mediated lipid trafficking, enhancement of astrocyte-mediated lipid buffering, and regulation of macrophage–hepatocyte communication have all shown promise in preclinical studies (Kaji et al., 2024). These approaches highlight the potential of targeting tissue-level metabolic cooperation as a complementary strategy for correcting dysfunction within the endo-lysosomal–lipid axis (Faust Akl and Quintana, 2026; Gegenhuber et al., 2026). Lipid turnover extends beyond individual organelles and individual cells. Efficient lipid clearance requires coordinated interactions among neurons, astrocytes, microglia, macrophages, hepatocytes, and other specialized cell populations. Therapeutic restoration of multicellular lipid clearance networks may represent an important future direction for the treatment of metabolic and neurodegenerative diseases (Figure 3).

## 6 Conclusions and future perspectives

The endo-lysosomal system has traditionally been viewed as a degradative network responsible for cargo turnover and intracellular waste disposal. However, accumulating evidence now indicates that it functions as a central regulatory hub that integrates membrane trafficking, lipid redistribution, nutrient sensing, autophagy, and inflammatory signaling. Throughout this review, we highlight how disturbances in these interconnected processes converge to form a dynamic endo-lysosomal–lipid axis that contributes to the development of metabolic and neurodegenerative diseases. Importantly, the relationship between lipid metabolism and endo-lysosomal function is not unidirectional. Lipid overload disrupts membrane organization, impairs endosomal maturation, destabilizes lysosomal acidification, and suppresses degradative capacity. In turn, lysosomal dysfunction promotes defective lipid trafficking, impaired cholesterol redistribution, autophagic failure, and secondary lipid accumulation. These reciprocal interactions generate self-reinforcing pathological circuits involving V-ATPase dysfunction, lysosomal acidification defects, impaired lipophagy, chronic inflammation, and metabolic stress. Rather than representing isolated cellular events, these processes collectively drive the progressive destabilization of cellular homeostasis. A major emerging theme is that the endo-lysosomal–lipid axis extends beyond individual organelles and even beyond individual cells. Neuronal compartmentalization creates a unique dependence on long-distance endo-lysosomal trafficking, rendering neurons particularly vulnerable to transport defects. At the tissue level, interactions among neurons, astrocytes, microglia, macrophages, and parenchymal cells coordinate lipid turnover and clearance, suggesting that metabolic dysfunction should increasingly be viewed as a multicellular phenomenon rather than a purely cell-autonomous process. Despite significant advances, several important questions remain unresolved. First, the spatial organization of endo-lysosomal trafficking pathways in different cellular compartments remains incompletely understood. Second, the mechanisms governing lipid exchange at MCSs and their integration with vesicle-mediated transport require further investigation. Third, the precise contribution of neuro-glial metabolic coupling to lipid homeostasis and disease progression remains largely unexplored. Addressing these questions will require the integration of emerging technologies, including spatial lipidomics, single-cell multi-omics, live-cell imaging, and *in situ* cryo-electron tomography. From a translational perspective, therapeutic strategies targeting individual metabolic pathways may be insufficient to restore homeostasis. Future interventions will likely need to simultaneously address lysosomal acidification, lipid trafficking, autophagic degradation, and intercellular lipid clearance. As our understanding of the endo-lysosomal–lipid axis continues to evolve, this integrated framework may provide new opportunities for the development of mechanism-based therapies for metabolic and neurodegenerative diseases.
